# 

**Published:** 1856-10

**Authors:** 


### CONTENTS.

ORIGINAL COMMUNICATIONS— PAGE.
Laryngotomy, after Apparent Death—Recovery. By George Amerman, M.D., late House Surgeon of Bellevue Hospital, ........ 439
Injury of the Knee-Joint — Inflammation — Amputation — Recovery. Treated by Dr. H. A. Johnson, one of the Physicians to the Mercy Hospital. Reported by P. J. Wardner. ........ 442
Poisoning by Strychnine—Case of William Palmer. By J. C. Morfit, M.D., Chi ago,. ........ 443
Singular Effect of a Blow on the Occupit. By the Editor, ........ 448
Medical Knowledge. By J. R. Freese, M.D., Bloomington, Ill., ........ 450
Case Bronchitis—Pleurisy, with Effusion—Pneumothorax—Death. By Ralph Isham, M.D., Chicago, ........ 452

SELECTIONS, ........
On the Alkaline Method of Treatment in Acute Rheumatism. By Jas. Graham, M.D., Professor of Materia Medica and Therapeutics, in the Medical College of Ohio,.. ........ 455
Ununited Fractures. Stated Meeting, New York Academy of Medicine, July 2, 1856. The President, Dr. W. Parker, in the Chair, ........ 458

BOOK NOTICES, ........
Physical Exploration and Diagnosis of Diseases Affecting the Respiratory Organs. By Austin Flint, M.D., Professor of the Theory and Practice of Medicine, in the University of Louisville, &c. ........ 463
A Practical Treatise on the Diseases of the Testis and of the Spermatic Cord and Scrotum, with numerous wood engravings. By F. B. Curling, F. R.S., Surgeon to the London Hospital, &c. ........ 474
Human Physiology. By Robley Dunglison, M.D., LL.D., &c., &c., &c. with five hundred and thirty-two Illustrations, ........ 475

EDITORIAL, ........
The Presidency of the American Medical Association, ........ 475
Dysentery, ........ 479
Rush Medical College, ........ 479
To Subscribers, ........ 480
Rush Medical College, ........ 480

MISCELLANEOUS ITEMS, ........
Mortality in the French Army in the East during the Late War, ........ 480
Cholera in Portugal and Madeira, ........ 482
Cure of Itch in Half an Hour, ........ 482
Alsidium Blodgettii in Consumption and Scrofulous Diseases, ........ 483
A New Instrument for Indicating the Movement of the Heart, ........ 484
Vaccination in Relation to Blindness, ........ 484
Bromine as a Specific in Pseudo-Membraneous Affections, ........ 485
Cholera in Moscow, ........ 485

